# Anticancer Effects of Sinocrassulosides VI/VII from* Silene viscidula* on HeLa Cells

**DOI:** 10.1155/2017/8240820

**Published:** 2017-07-09

**Authors:** Hang Chen, Qian Ma, Wei Xu, Wan-Ming Li, De-Zheng Yuan, Jia-Mei Wu, Yu-Shan Li, Jin Fang

**Affiliations:** ^1^Department of Cell Biology, Key Laboratory of Cell Biology, Ministry of Public Health and Key Laboratory of Medical Cell Biology, Ministry of Education, China Medical University, No. 77 Puhe Road, Shenyang North New Area, Shenyang 110122, China; ^2^School of Traditional Chinese Materia Medica, Shenyang Pharmaceutical University, Wenhua Road 103, Shenhe District, Shenyang 110016, China

## Abstract

Natural products are becoming increasingly important in chemoprevention and for cancer therapy.* Silene viscidula (S. viscidula)*, a traditional Chinese herb, has long been used as an anti-inflammatory and neuroleptic agent. However, the anticancer activity of* S. viscidula* has remained unclear. In this study, 16 compounds were extracted from* S. viscidula*. Among those compounds, sinocrassulosides VI/VII, an inseparable isomer mixture, possess the strongest inhibitory activity on HeLa cells with the IC_50_ value of 2.37 *μ*M. Mechanism studies found that sinocrassulosides VI/VII downregulated the expression of cyclin D1 and decreased retinoblastoma (Rb) phosphorylation, which arrested HeLa cells in the G1 phase. Also, sinocrassulosides VI/VII could induce senescence via the upregulation of p16 and a significant increase of *β*-galactosidase (*β*-gal) staining. Our results suggest that sinocrassulosides VI/VII may be a new therapeutic potential agent for cervical cancer. In addition, we explored the structure-activity relationships of three groups of the configurational isomer with similar chemical structure from* S. viscidula*. We first demonstrated that the length of the ester chains linked to the carboxyl group of the glucuronic acid residue could affect the potent cytotoxicity. This finding will open new avenues for developing effective anticancer compounds by modifying the components derived from plants in nature.

## 1. Introduction

The past two decades have seen the widespread use of natural products as cancer therapeutic and chemopreventive agents [1–3]. Some products purified from plants have been developed as effective drugs for cancer treatment. For example, paclitaxel (Taxol), a natural product from the bark of the Pacific yew, is used widely as chemotherapeutic drug against many forms of cancer [4, 5]. Moreover, some more effective drugs have been developed by structurally modifying natural products. C-28 methyl ester of 2-cyano-3,12-dioxooleana-1,9(11)-dien-28-oicacid (CDDO-Me), synthesized by structural modification of oleanolic acid, has been used in phase I studies in advanced solid tumors and lymphomas patients [6, 7]. Therefore, the development of new anticancer drugs from natural products especially plants is an important strategy of cancer treatment.

The roots of* Silene viscidula* Franch.* (S. viscidula)* are called “Wacao” and have been used as a traditional herbal medicine in the treatment of a wide range of diseases, including cough, gonorrhea, rheumatism, and bone pain in the southwestern region of China [8]. However, no previous study has sought to investigate the anticancer effect of* S. viscidula.* In this study, we isolated and identified 16 triterpenoid saponins from EtOH extract of the dried roots of* S. viscidula*. Triterpenoid saponins have been proven as potential agents for the therapy of cancer [9–11]. Thus, we aimed to investigate whether triterpenoid saponins from* S. viscidula* have anticancer activities.

We first detected the cytotoxicity of 16 compounds isolated from the roots of* S. viscidula*. Sinocrassulosides VI/VII (compounds 12/13), an inseparable isomer mixture, showed the most potent growth inhibition for different types of cancer cells. Sinocrassulosides VI and VII are oleanane-type triterpenoid saponins and were first isolated from* Sinocrassula asclepiadea* [12] by Zhao et al. in 2004. Although some studies have demonstrated that oleanane-type triterpenoids could inhibit the proliferation of tumor cells by inducing cell cycle arrest as well as apoptosis [13–15], there have been no reports on anticancer activities of sinocrassulosides VI and VII up to now.

In this study, we investigated the anticancer mechanism of sinocrassulosides VI/VII using the human cervical cancer cell line HeLa whose growth was inhibited more strongly compared with eight other cancer cell lines detected. In addition, we also compared the anticancer activities of three groups of the configuration isomer, including viscidulosides A/B, sinocrassulosides VI/VII, and sinocrassulosides VIII/IX, and first found the possible relationship between anticancer activity and glucuronic acid residue at the sugar chain of the C-3 position of oleanane-type triterpenoid saponins. Our study provides an important clue for discovering and developing new effective components for cancer therapy.

## 2. Materials and Methods

### 2.1. Cell Line Culture Conditions and Reagents

The cell lines used included the human colorectal cancer cell lines SW620 and HT-29, the human gastric cancer cell lines SGC-7901 and BGC823, the human breast cancer cell lines MCF-7 and MDA-MB-435, the human lung cancer cell line A549, the human glioblastoma cancer cell line U87MG, and the human cervical cancer cell line HeLa. All of the cells were cultured at 37°C under a 5% CO_2_ atmosphere. SW620, HT-29, and A549 were grown in RPMI-1640 supplemented with 10% FBS and 100 units/mL penicillin-streptomycin. The growth medium for the other cells was Dulbecco's modified Eagle medium (DMEM) supplemented with 10% FBS and 100 units/mL penicillin-streptomycin.

### 2.2. Extraction and Isolation of Effective Components from* S. viscidula*

**Figure 1:**
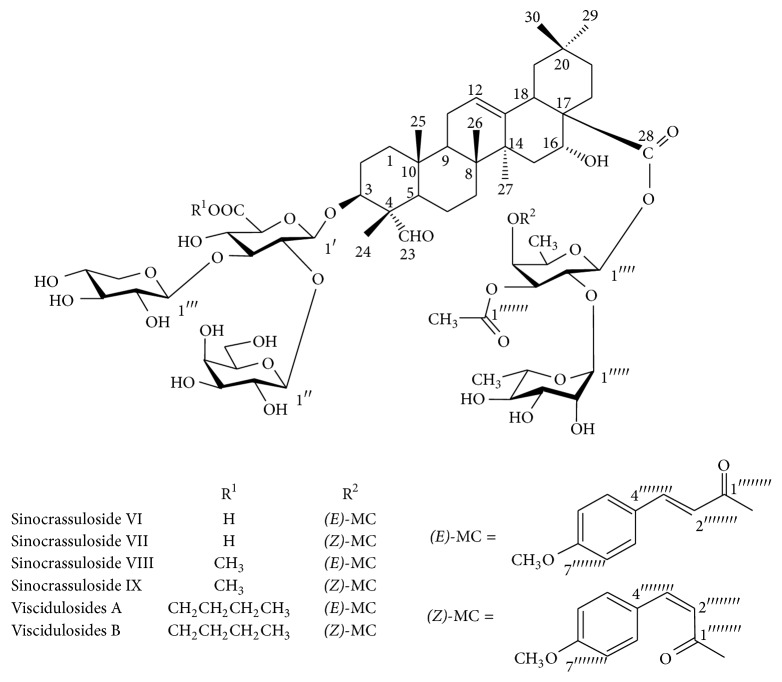
The chemical structures of sinocrassulosides VI/VII (12/13), sinocrassulosides VIII/IX (14/15), and viscidulosides A/B (7/8) from* S. viscidula*.

Sixteen compounds from* S. viscidula* were isolated as previously described [16, 17]. Briefly, the dried roots of* S. viscidula* were extracted with 70% ethanol. The EtOH extract was suspended in H_2_O and extracted with petroleum ether, AcOEt, and BuOH, successively. The BuOH soluble extract was further subjected to silica gel column chromatography (10 × 100 cm) to yield fractions. Finally, 16 single components were obtained, including silenoviscoside D (4), silenoviscoside F (6), visciduloside A (7), visciduloside B (8), sinocrassulosides II, Ι, and VI~IX (9, 10, 12~15), dianchinenoside D (11), oleanolic acid (16) [16, 17], and four unpublished compounds (1, 2, 3, and 5), in which viscidulosides A (7)/B (8), sinocrassulosides VI (12)/VII (13), and sinocrassulosides VIII (14)/IX (15) were three inseparable mixtures, which are glycosides of quillaic acid whose fucose residue is acylated by a* (E)*- or* (Z)*-4-methoxycinnamic acid (Figure 1). The sinocrassulosides VI/VII were identified by comparing their spectral data (Figures S1–S3, Tables S1-S2 in Supplementary Material available online at https://doi.org/10.1155/2017/8240820) with those in the literature [12]. Sixteen compounds from* S. viscidula* were dissolved in dimethyl sulfoxide (DMSO) for storage, and working solution was produced by dilution of stock solution using cell culture medium with a final DMSO concentration of less than 0.1%.

### 2.3. Cell Viability Assay

Growth inhibition effects of the compounds from* S. viscidula* on cancer cells were measured by MTT assay (Promega, Madison, WI, USA). Briefly, cells were collected and seeded in 96-well plates at a density of 5 × 10^3^ cells and treated with 100 *μ*M compounds extracted from* S. viscidula* for 24 h, while cell culture medium with 0.1% DMSO was used as a control. Then, the cells were washed twice with PBS and incubated with 10 *μ*l of MTT (5 mg/mL) for 4 h at 37°C. Afterwards, the medium was removed, and 200 *μ*l of DMSO was added to each well to solubilize the formazan crystals. The plates were incubated at room temperature for 15 min and the absorbance at 595 nm was read by a microplate reader (BIO-RAD 680, USA). The percent of growth inhibition was calculated as (OD of control − OD of the treated group)/(OD of the control group − OD of blank) × 100. The CCK-8 assay (Beyotime, Jiangsu, China) was performed to measure the growth inhibition of compounds on HeLa cells. HeLa cells (5 × 10^3^/well) were cultured in 96-well plates and then treated with sinocrassulosides VI/VII (1, 2, 3, 4, and 5 *μ*M) for 12 or 24 h and sinocrassulosides VIII/IX and viscidulosides A/B (6, 8, 10, 20, and 30 *μ*M) for 24 h at 37°C. After incubation, 10 *μ*l of CCK-8 was added to the culture medium and incubated for an additional 2 h at 37°C. The absorbance was read at the wavelength of 450 nm. Percent of growth inhibition was calculated as described above.

In addition, the cells were seeded in 96-well plates to further observe the cellular morphology through phase-contrast microscopy (Nikon TMS, Japan). The fluorescent images of the cells were obtained through fluorescence microscopy (Olympus IX51, Japan). The cells were treated with different concentrations of sinocrassulosides VI/VII for 12 h, fixed with 4% formaldehyde for 15 min, and then stained with DAPI for 5 min to counterstain the nucleus.

### 2.4. Cell Cycle Analysis

HeLa cells were harvested, exposed to sinocrassulosides VI/VII at different concentrations (2, 4, and 8 *μ*M) for 12 h, then digested by trypsinization, washed with ice-cold PBS, and fixed in 70% ethanol at 4°C overnight. The cells were washed and incubated with 400 *μ*l of propidium iodide (PI) (Sangon, Shanghai, China) in the dark at room temperature for 30 min. The cell cycle was analyzed by flow cytometry (Becton Dickinson, San Jose, CA).

### 2.5. Western Blotting and Immunoprecipitation

HeLa cells were treated with different concentrations of sinocrassulosides VI/VII (2, 4, and 8 *μ*M) for 12 h at 37°C. Cells were lysed using ice-cold modified RIPA buffer [50 mM Tris-HCl, 150 mM NaCl, 0.25% SDS, 1% Triton X-100, 0.25% sodium-deoxycholate, 1 mM EDTA, 1 mM EGTA, 1 mM dithiothreitol (DTT), and protease inhibitor cocktail (Roche)]. Proteins from the cell lysate were separated by SDS-PAGE and transferred to PVDF membranes (Millipore, Billerica, USA). The membranes were incubated with primary antibodies p16, CDK4, CDK6, cyclinD1, pRb, E2F1, and Tubulin (1 : 1000, Cell Signaling Technology, Beverly, USA) overnight at 4°C, followed by incubation with HRP-conjugated secondary antibodies (1 : 5000, Santa Cruz, CA) for 1 h at room temperature. All signals were visualized using ECL Western blotting substrate (Pierce, Thermo Fisher Scientific) according to the instructions of the manufacturer.

For immunoprecipitation, HeLa cells were treated with 4 *μ*M sinocrassulosides VI/VII at 37°C for 12 h. Cell protein extracts (1 mg protein in 500 *μ*l lysis buffer) were incubated with 2 *μ*g of Rb antibodies or normal IgG at 4°C for 2 h, followed by 50 *μ*l of protein G-agarose suspension (Santa Cruz, CA) overnight at 4°C with gentle shaking. The immunocomplexes were washed three times with fresh RIRA buffer and eluted by boiling the samples in 2x SDS-PAGE loading buffer. The proteins were electrophoresed on SDS-PAGE and transferred to PVDF membranes. The membranes were incubated with E2F1 antibodies (0.5 *μ*g/mL) overnight at 4°C, followed by incubation with HRP-conjugated secondary antibodies for 1 h at room temperature. All signals were visualized using ECL Western blotting substrate.

### 2.6. Senescence-Associated *β*-Gal Activity Assay

Senescence was assessed using a senescence *β*-gal staining kit (Beyotime, Jiangsu, China) following the manufacturer's protocol. Briefly, HeLa cells were cultured on 100 mm^2^ dishes and then pretreated with sinocrassulosides VI/VII (4 *μ*M) for 12 h at 37°C. Cells were fixed and incubated with freshly prepared *β*-gal staining solution at 37°C overnight. *β*-gal staining cells were detected under a microscope.

### 2.7. Statistical Analysis

The data were expressed as mean ± standard deviation (SD) of three independent experiments. The statistical analysis was done with Student's* t*-test. A two-tailed *p* value of <0.05 was considered statistically significant.

## 3. Results

### 3.1. Sinocrassulosides VI/VII Inhibited Proliferation of Tumor Cells

**Figure 2:**
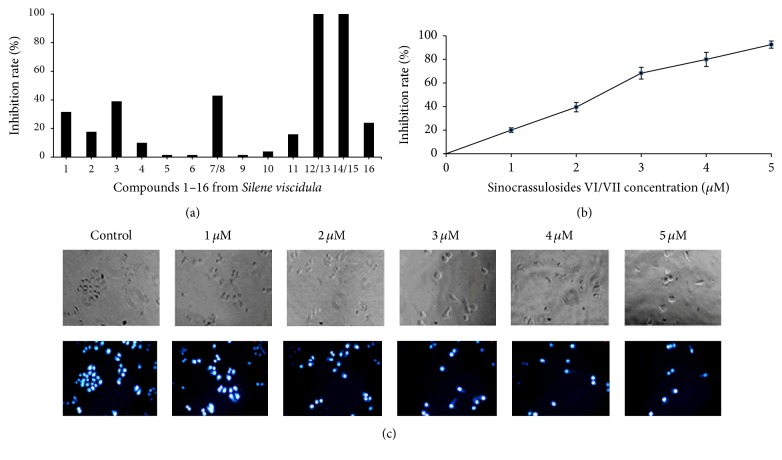
Proliferation inhibition effects of the compounds from* S. viscidula* on cancer cells. (a) HT-29 cells were treated with compounds 1–16 from* S. viscidula* for 24 h. The cell viability was then measured by MTT assays. (b) HeLa cells were treated with different concentrations of sinocrassulosides VI/VII for 12 h. The CCK-8 assays were performed. Data were presented as mean ± SD of three independent experiments. (c) HeLa cells were treated with different concentrations of sinocrassulosides VI/VII for 12 h and observed under a phase-contrast microscope. Magnification: ×100. DAPI counterstaining was analyzed by fluorescence microscopy.

To determine the cytotoxic effects of* S. viscidula* on cancer cells, we first investigated the inhibition effects of 16 compounds from* S. viscidula* on the proliferation of HT-29 cells. MTT assay showed that five compounds of them could inhibit cell proliferation at the concentration of 100 *μ*M. Sinocrassulosides VI/VII (12/13) and sinocrassulosides VIII/IX (14/15) showed a 100% growth inhibition (Figure 2(a)). Because sinocrassulosides VI/VII exhibited a stronger antiproliferative effect on HT-29 cells than that of sinocrassulosides VIII/IX at the concentration of 40 *μ*M (data not shown), we next focused on investigating the growth inhibition of sinocrassulosides VI/VII on nine types of cancer cell lines, including SW620, SGC-7901, BGC823, MCF-7, HeLa, HT-29, MDA-MB-435, A549, and U87MG. After sinocrassulosides VI/VII treatment, proliferation of nine cell lines was inhibited with different IC_50_ values of 2–5 *μ*M, among which HeLa cell was the most sensitive to sinocrassulosides VI/VII with the IC_50_ value of 2.37 *μ*M (Figure S4). Therefore, it was selected as the model for investigating the anticancer mechanisms of sinocrassulosides VI/VII.

**Figure 3:**
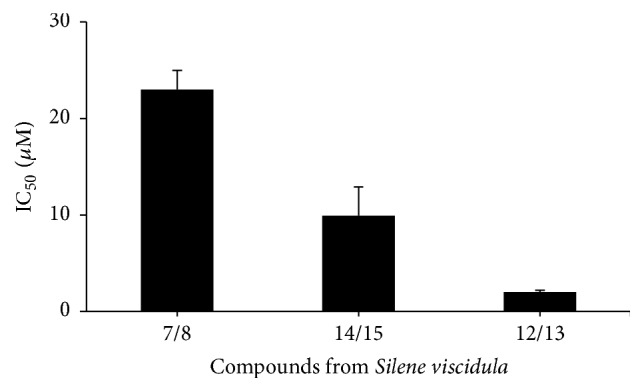
Growth inhibition of HeLa cells by sinocrassulosides VI/VII (12/13), sinocrassulosides VIII/IX (14/15), and viscidulosides A/B (7/8) from* S. viscidula*. HeLa cells were treated by different concentrations of sinocrassulosides VI/VII (1, 2, 3, 4, and 5 *μ*M) or sinocrassulosides VIII/IX and viscidulosides A/B (6, 8, 10, 20, and 30 *μ*M) for 24 h. The cell viability was then measured by CCK-8 assays. Data are mean ± SD of three independent experiments.

In CCK-8 assay, sinocrassulosides VI/VII were detected for the cytotoxic effect on HeLa cells at various concentrations ranging from 1 *μ*M to 5 *μ*M for 12 h.  Figure 2(b) shows that sinocrassulosides VI/VII inhibit cell proliferation in a dose-dependent manner. Consistently, under a phase-contrast microscope, sinocrassulosides VI/VII led to a dose-dependent decrease in cell numbers, further indicating the growth inhibition effect of sinocrassulosides VI/VII (Figure 2(c)). In addition, we compared the anticancer activity of three groups of conformer compounds with similar chemical structure using HeLa cells. The order of the cytotoxicity in terms of IC_50_ values was viscidulosides A/B > sinocrassulosides VIII/IX > sinocrassulosides VI/VII (Figure 3).

### 3.2. Sinocrassulosides VI/VII Inhibited the Proliferation of Hela Cells by Inducing a G1 Phase Arrest

**Figure 4:**
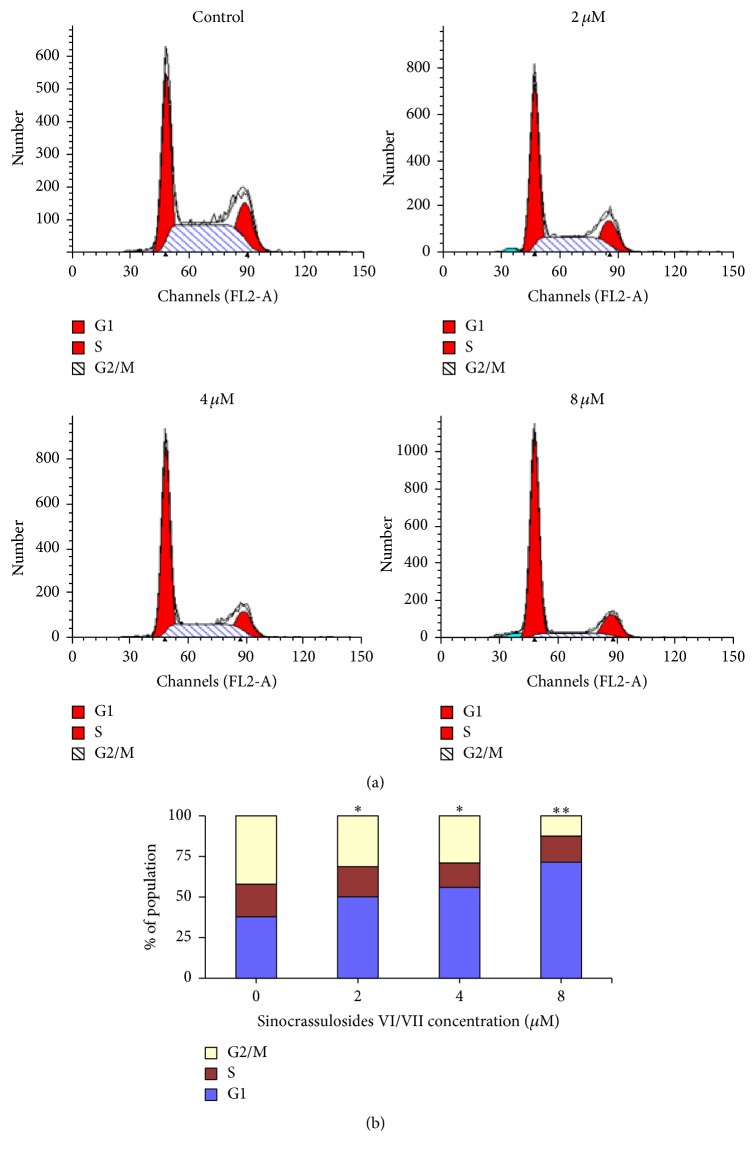
Sinocrassulosides VI/VII induced the accumulation of HeLa cells in G1 phase. The cell cycle distribution was analyzed by flow cytometry. The representative graphs are shown in (a). The quantitative analysis is demonstrated as histograms in (b). Data are mean ± SD of three independent experiments. ^*∗*^*p* < 0.05; ^*∗∗*^*p* < 0.01.

**Figure 5:**
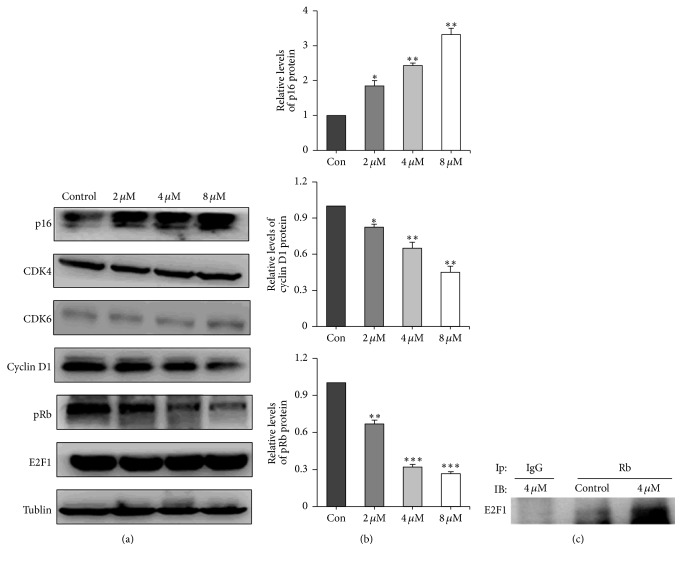
Sinocrassulosides VI/VII regulated the expression of cell cycle regulators in HeLa cells. (a) The expression of p16, CDK4, CDK6, cyclin D1, pRb, and E2F1 was detected by Western blot analysis. Tubulin was used as a loading control. (b) The quantitative analysis of p16, cyclin D1, and pRb proteins was illustrated as histograms. Data are mean ± SD of three independent experiments. Asterisks show significant difference compared to the control group (^*∗*^*p* < 0.05, ^*∗∗*^*p* < 0.01, ^*∗∗∗*^*p* < 0.001). (c) Cell lysates were subjected to immunoprecipitation using anti-Rb or normal IgG antibodies. The immunoprecipitated products were immunoblotted with anti-E2F1 antibodies.

To investigate the mechanism by which sinocrassulosides VI/VII inhibited HeLa cell proliferation, we performed cell cycle analysis. HeLa cells were treated with different concentrations of sinocrassulosides VI/VII (2, 4, and 8 *μ*M). Cytometry analysis showed that sinocrassulosides VI/VII caused a dose-dependent accumulation of cells in the G1 phases compared to untreated cells (Figure 4). Further, we assessed the expression of cell cycle related proteins, including cyclin D1, CDK4, CDK6, p16, pRb, and E2F1 in HeLa cells. As shown in Figures 5(a) and 5(b), after treatment with 2, 4, and 8 *μ*M of sinocrassulosides VI/VII, the protein levels of p16 increase, and the expression of cyclin D1 and pRb decreases in a dose-dependent manner. However, the expression of CDK4, CDK6, and E2F1 showed no significant change compared with the control. Also, we investigated Rb-E2F1 complexes' change by immunoprecipitation assay (Ip). Ip showed that the amount of E2F1 precipitated by the Rb antibodies was significantly increased (Figure 5(c)), indicating that the levels of the complex were enhanced.

### 3.3. Sinocrassulosides VI/VII Induced Senescence in HeLa Cells

**Figure 6:**
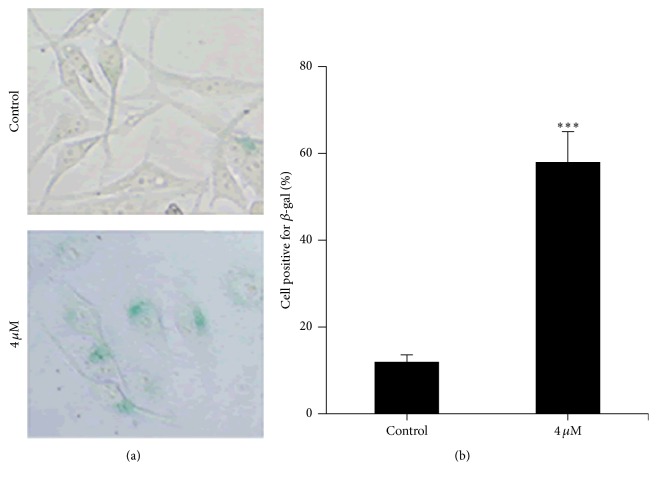
Sinocrassulosides VI/VII induced senescence in HeLa cells. *β*-Gal staining was performed to detect senescence cells. (a) The representative graphs are shown, magnification ×400. (b) *β*-Gal activity was quantified by counting 100 cells on three separate areas for each treatment. Asterisk indicates a statistically significant difference from control group (^*∗∗∗*^*p* < 0.001).

The increase of p16 level could be indicative of the activation of a cellular senescence program [18]. We then investigated whether sinocrassulosides VI/VII could induce senescence in HeLa cells. *β*-Gal staining, a specific marker of senescence cells, was measured. As shown in Figure 6(a), sinocrassulosides VI/VII-treated cells display blue stained cytoplasm, whereas untreated cells have no detectable blue staining. The percentage of *β*-gal staining positive cells was significantly increased up to 55% in comparison with 8% in the control group (Figure 6(b)), which suggests that sinocrassulosides VI/VII induced senescence in HeLa cells.

## 4. Discussion

In this study, EtOH extracts of* S. viscidula* were first reported to exert anticancer activities. In 16 compounds, sinocrassulosides VI/VII exhibited potent cytotoxicity against HeLa cells with the IC_50_ value of 2.37 *μ*M, indicating a strong anticancer activity, which may provide a new source for developing effective drugs for cancer treatment. Sinocrassulosides VI/VII also could significantly inhibit the growth of eight other cancer cells including colorectal, gastric, breast, lung, and glioblastoma, revealing a broad-spectrum antiproliferative activity.

Furthermore, we explored the anticancer mechanism of sinocrassulosides VI/VII. Flow cytometry assay demonstrated that sinocrassulosides VI/VII induced G1 phase arrest in HeLa cells. In G1 phase cells, the kinase activity of CDK4/6 is activated by binding to cyclin D1 and forming a complex, which can lead to Rb phosphorylation. Transcription factor E2F1 is released by pRb and thus promotes the transcription of E2F1 downstream genes, leading to the G1 to S transition [19, 20]. In this study, the level of cyclin D1 protein was decreased, and the expression of p16 was upregulated after sinocrassulosides VI/VII treatment. As an inhibitor of CDKs, p16 could prevent the formation of the cyclin-CDK 4/6 complexes [21, 22]. Our results showed that either the downregulation of cyclin D1 or the overexpression of p16 inactivated CDK4/6 and led to a pRb decrease. Furthermore, we found that the level of the Rb-E2F1 complex was increased after sinocrassulosides VI/VII treatment, which inhibited the release of E2F1 and, in turn, resulted in cell growth inhibition.

Growth arrest may be associated with apoptosis or senescence of cells [23, 24]. Identified as a senescence marker, p16 inhibits CDK4/6-mediated Rb phosphorylation and leads to an irreversible cell cycle arrest [25, 26]. Here, we observed that a fraction of cells displayed a flattened, enlarged morphology after sinocrassulosides VI/VII treatment. The overexpression of p16 and the increased number of *β*-gal staining cells supported the evidence that sinocrassulosides VI/VII induced senescence in HeLa cells. Triterpenoid saponins have been proven as potential antitumor agents, due to their ability to induce apoptosis in cancer cells [27, 28]. We are the first to demonstrate that oleanane-type triterpenoid saponin from* S. viscidula* may inhibit cell proliferation by inducing cell cycle arrest and senescence besides the reported apoptosis mechanism.

All 16 compounds isolated from* S. viscidula*, except for compounds 6 and 16, belong to oleanane-type pentacyclic triterpenoid saponins, indicating that this structure may be one of the main active components of the plant. Among 16 compounds, sinocrassulosides VI/VII, sinocrassulosides VIII/IX, and viscidulosides A/B, sharing common structural features of a CHO substituent at C-23 and a* p*-methoxycinnamoyl group of fucose residue at C-28 sugar chain (Figure 1), exhibited strong anticancer activity, while other compounds, either with a COOH not CHO substituent at C-23 or without* p*-methoxycinnamoyl at C-28 sugar chain, showed less or no cytotoxic activity. This finding is consistent with a previous study that the ideal anticancer saponins are of an oleanane type with the presence of sugar residues linked to the C-28 position [29]. Moreover, we found that the carboxyl group of the glucuronic acid at C-3 sugar chain could determine the cytotoxic activity of the three compounds. Sinocrassulosides VI/VII with a free carboxyl group showed the strongest cytotoxic activity, while sinocrassulosides VIII/IX with a methyl linked to the carboxyl group showed higher cytotoxicity than viscidulosides A/B with the presence of a butyl attached chain at the carboxyl group. These results suggest that their cytotoxicity decreased with the longer ester chain linked to the carboxyl group of the glucuronic acid at C-3, indicating that glucuronic acid residue at C-3 sugar chain might be the anticancer active group of these compounds. Zhang et al.'s study on the evaluation of saponins as immunomodulators found that the hydrophobicity of the ester chain bonds to the carboxyl group of the glucuronic acid residue at C-3 of the triterpenoid saponins could affect hemolytic activity and adjuvant potentials [30]. In this study, we demonstrate that decreasing the length of ester chain bonds to the carboxyl group could enhance anticancer activity, further revealing that the glucuronic acid residue at C-3 sugar chain is an important reaction site for modifying bioactivities of saponins.

## 5. Conclusions

Our studies demonstrated for the first time that* S. viscidula* has anticancer activities. Sinocrassulosides VI/VII possess the strongest anti-cervical cancer activity through the induction of cell cycle arrest and senescence, indicating that sinocrassulosides VI/VII are potentially novel, natural anticancer compounds. In addition, the structure-activity relationships' analysis revealed that the carboxyl group of glucuronic acid at C-3 sugar chain determines the cytotoxic activity, which could provide clues for the synthesis of oleanolic-type triterpenoid saponin derivatives.

## Supplementary Material

The spectral data were provided in Figures S1–S3 and Tables S1-S2 for identification of the sinocrassulosides VI/VII. In addition, Figure S4 shows the effects of sinocrassulosides VI/VII on nine human cancer cell lines.
