# Supplementary material for: Anticancer Effects of Sinocrassulosides VI/VII from Silene viscidula on HeLa Cells
Source: Evid Based Complement Alternat Med. 2017 Jul 9;2017:8240820. doi: 10.1155/2017/8240820 (PMC5523537; doi:10.1155/2017/8240820)
Supplement: Supplementary file 1 — The spectral data were provided in Figures S1–S3 and Tables S1-S2 for identification of the sinocrassulosides VI/VII. In addition, Figure S4 shows the effects of sinocrassulosides VI/VII on nine human cancer cell lines. [file 8240820.f1.docx]

**Supplementary Material**

**Anticancer Effects of Sinocrassulosides VI/VII from *Silene Viscidula* on HeLa Cells**

**Hang Chen, ^1^ Qian Ma, ^1^ Wei Xu, ^2^ Wan-Ming Li,^1^ De-Zheng Yuan, ^1^ Jia-Mei Wu, ^1^ Yu-Shan Li ,^2^ Jin Fang,^1^**

*^1^* *Department of Cell Biology, Key Laboratory of Cell Biology, Ministry of Public Health, and Key Laboratory of Medical Cell Biology, Ministry of Education, China Medical University,* *No.77 Puhe Road, Shenyang North New Area, Shenyang 110122, China*

*^2^ School of Traditional Chinese Materia Medica, Shenyang Pharmaceutical University,* *Wenhua Road 103, Shenhe District, Shenyang 110016, China*

Correspondence should be addressed to Jin Fang; E-mail: jfang61@netease.com

## Supplementary Figures

**Figure S1**





**FIGURE S1. ESIMS (positive) spectrum of sinocrassulosides VI/VII**

**Figure S2**

**

**

**FIGURE S2. ^1^H NMR spectrum of sinocrassulosides VI/VII**

**Figure S3**





**FIGURE S3. ^13^C NMR spectrum of sinocrassulosides VI/VII**

**Figure S4**





**FIGURE S4.** **Effect of sinocrassulosides VI/VII from *S. viscidula* on nine human cancer cell lines**. Nine cancer cell lines were treated by sinocrassulosides VI/VII at the indicated final concentrations (1, 2, 3, 4 and 5 μM). The MTT assay was performed. Data were presented as mean ± S.D. of three independent experiments.

**Table S1. ^13^C-NMR spectral data (150 MHz, *δ* in ppm, *J* in Hz) of sinocrassulosides VI/VII in CD_3_OD**

| Position | sinocrassulosides VI | sinocrassulosides VII | Position | sinocrassulosides VI | sinocrassulosides VII |
| --- | --- | --- | --- | --- | --- |
|  | The triterpenoid moiety | | 2'' | 74.2 | 74.2 |
| 1 | 39.4 | 39.4 | 3'' | 75.5 | 75.5 |
| 2 | 25.8 | 25.8 | 4'' | 70.9 | 70.9 |
| 3 | 86.5 | 86.5 | 5'' | 76.9 | 76.9 |
| 4 | 56.0 | 56.1 | 6'' | 62.4 | 62.4 |
| 5 | 48.7 | 48.9 |  | 3'-*O*-β-d-Xyl | |
| 6 | 21.0 | 21.0 | 1''' | 105.1 | 105.1 |
| 7 | 31.9 | 32.0 | 2''' | 75.3 | 75.3 |
| 8 | 41.3 | 41.3 | 3''' | 78.3 | 78.3 |
| 9 | 48.1 | 48.1 | 4''' | 70.9 | 70.9 |
| 10 | 36.6 | 36.6 | 5''' | 67.3 | 67.3 |
| 11 | 24.7 | 24.8 |  | 28-*O*-β-d-Fuc | |
| 12 | 123.4 | 123.4 | 1'''' | 95.1 | 95.2 |
| 13 | 144.8 | 144.8 | 2'''' | 72.1 | 72.1 |
| 14 | 43.0 | 43.0 | 3'''' | 75.1 | 75.1 |
| 15 | 36.6 | 36.6 | 4'''' | 71.5 | 71.5 |
| 16 | 73.8 | 73.8 | 5'''' | 71.1 | 71.1 |
| 17 | 50.4 | 50.3 | 6'''' | 16.6 | 16.6 |
| 18 | 42.5 | 42.7 |  | 2''''-*O*-α-l-Rha | |
| 19 | 48.1 | 48.1 | 1''''' | 102.5 | 102.7 |
| 20 | 30.9 | 30.9 | 2''''' | 71.8 | 71.8 |
| 21 | 37.2 | 37.2 | 3''''' | 72.0 | 72.0 |
| 22 | 31.5 | 31.5 | 4''''' | 73.7 | 73.7 |
| 23 | 210.0 | 210.0 | 5''''' | 71.1 | 71.1 |
| 24 | 11.0 | 11.0 | 6''''' | 18.5 | 18.5 |
| 25 | 16.5 | 16.6 |  | 3''''-*O*-Acetyl group | |
| 26 | 18.2 | 18.3 | 1'''''' | 171.7 | 171.7 |
| 27 | 27.4 | 27.4 | 2'''''' | 21.1 | 21.1 |
| 28 | 177.2 | 177.2 |  | 4''''-*O*-MC | |
| 29 | 33.6 | 33.6 | 1''''''' | 168.5 | 167.6 |
| 30 | 25.4 | 25.3 | 2''''''' | 115.5 | 116.6 |
|  | 3-*O*-β-d-Gul-A | | 3''''''' | 147.4 | 146.1 |
| 1' | 103.9 | 103.9 | 4''''''' | 128.3 | 128.7 |
| 2' | 78.2 | 78.2 | 5''''''' | 131.4 | 133.8 |
| 3' | 86.5 | 86.5 | 6''''''' | 115.7 | 114.7 |
| 4' | 71.1 | 71.1 | 7''''''' | 163.6 | 162.5 |
| 5' | 76.4 | 76.4 | 8''''''' | 115.7 | 114.7 |
| 6' | 170.9 | 170.9 | 9''''''' | 131.4 | 133.8 |
|  | 2'-*O*-β-d-Gal | | 7'''''''-OCH_3_ | 56.1 | 56.0 |
| 1'' | 104.6 | 104.6 |  |  |  |

**Table S2 ^1^H NMR spectral data of sinocrassulosides VI/VII (CD_3_OD, 600 MHz).**

| Position | **12** | **13** | Position | **12** | **13** |
| --- | --- | --- | --- | --- | --- |
|  | *δ*_H_ (*J* = Hz) | *δ*_H_ (*J* = Hz) |  | *δ*_H_ (*J* = Hz) | *δ*_H_ (*J* = Hz) |
| 3 | 3.92 (1H, *m*) | 3.97 (1H, *m*) | 1''' | 4.42 (1H, *d*, 7.2) | 4.40 (1H, *d*, 7.2) |
| 12 | 5.32 (1H, *br s*) | 5.38 (1H, *br s*) | 1'''' | 5.51 (1H, *d*, 7.8) | 5.49 (1H, *d*, 7.8) |
| 16 | 4.48 (1H, *m*) | 4.48 (1H, *m*) | 6'''' | 1.13 (3H, *d*, 8.4) | 1.12 (3H, *d*, 8.4) |
| 18 | 2.98 (1H, *dd*, 14.2, 2.5) | 2.95 (1H, *dd*, 14.2, 2.5) | 1''''' | 4.96 (1H, *br s*) | 4.93 (1H, *br s*) |
| 23 | 9.42 (1H, *s*) | 9.43 (1H, *s*) | 6''''' | 1.22 (3H, *d*, 6.0) | 1.23 (3H, *d*, 6.0) |
| 24 | 1.13 (3H, *s*) | 1.12 (3H, *s*) | 2'''''' | 2.04 (3H, *s*) | 2.00 (3H, *s*) |
| 25 | 1.00 (3H, *s*) | 1.00 (3H, *s*) | 2''''''' | 6.49 (1H, *d*, 16.2) | 5.93 (1H, *d*, 12.6) |
| 26 | 0.81 (3H, *s*) | 0.80 (3H, *s*) | 3''''''' | 7.71 (1H, *d*, 15.6) | 6.99 (1H, *d*, 12.6) |
| 27 | 1.40 (3H, *s*) | 1.39 (3H, *s*) | 5''''''' | 7.60 (1H, *d*, 8.4) | 7.77 (1H, *d*, 9.0) |
| 29 | 0.91 (3H, *s*) | 0.90 (3H, *s*) | 6''''''' | 6.97 (1H, *d*, 8.4) | 6.92 (1H, *d*, 8.4) |
| 30 | 0.99 (3H, *s*) | 0.97 (3H, *s*) | 8''''''' | 6.97 (1H, *d*, 8.4) | 6.92 (1H, *d*, 8.4) |
| 1' | 4.59 (1H, *d*, 7.8) | 4.58 (1H, *d*, 7.8) | 9''''''' | 7.60 (1H, *d*, 8.4) | 7.77 (1H, *d*, 9.0) |
| 1'' | 4.80 (1H, *d*, 6.6) | 4.80 (1H, *d*, 6.6) | 7'''''''-OCH_3_ | 3.83 (3H, *s*) | 3.85 (3H, *s*) |
